# Spondylolysis and spinal adaptations for bipedalism: The overshoot hypothesis

**DOI:** 10.1093/emph/eoaa003

**Published:** 2020-03-03

**Authors:** Kimberly A Plomp, Keith Dobney, Mark Collard

**Affiliations:** e1 Department of Archaeology, Simon Fraser University, 8888 University Drive, Burnaby, BC V5A 1S6, Canada; e2 Department of Archaeology, Classics and Egyptology, University of Liverpool, 14 Abercromby Square, Liverpool L69 7WZ, UK; e3 Department of Archaeology, University of Aberdeen, St Mary’s, Elphinstone Road, Aberdeen AB24 3UF, UK

**Keywords:** vertebrae, spinal pathology, Schmorl’s nodes, chimpanzee, gorilla, orangutan

## Abstract

**Background and objectives:**

The study reported here focused on the aetiology of spondylolysis, a vertebral pathology usually caused by a fatigue fracture. The goal was to test the Overshoot Hypothesis, which proposes that people develop spondylolysis because their vertebral shape is at the highly derived end of the range of variation within *Homo sapiens*.

**Methodology:**

We recorded 3D data on the final lumbar vertebrae of *H. sapiens* and three great ape species, and performed three analyses. First, we compared *H. sapiens* vertebrae with and without spondylolysis. Second, we compared *H. sapiens* vertebrae with and without spondylolysis to great ape vertebrae. Lastly, we compared *H. sapiens* vertebrae with and without spondylolysis to great ape vertebrae and to vertebrae of *H. sapiens* with Schmorl’s nodes, which previous studies have shown tend to be located at the ancestral end of the range of *H. sapiens* shape variation.

**Results:**

We found that *H. sapiens* vertebrae with spondylolysis are significantly different in shape from healthy *H. sapiens* vertebrae. We also found that *H. sapiens* vertebrae with spondylolysis are more distant from great ape vertebrae than are healthy *H. sapiens* vertebrae. Lastly, we found that *H. sapiens* vertebrae with spondylolysis are at the opposite end of the range of shape variation than vertebrae with Schmorl’s nodes.

**Conclusions:**

Our findings indicate that *H. sapiens* vertebrae with spondylolysis tend to exhibit highly derived traits and therefore support the Overshoot Hypothesis. Spondylolysis, it appears, is linked to our lineage’s evolutionary history, especially its shift from quadrupedalism to bipedalism.

Lay summary: Spondylolysis is a relatively common vertebral pathology usually caused by a fatigue fracture. There is reason to think that it might be connected with our lineage’s evolutionary shift from walking on all fours to walking on two legs. We tested this idea by comparing human vertebrae with and without spondylolysis to the vertebrae of great apes. Our results support the hypothesis. They suggest that people who experience spondylolysis have vertebrae with what are effectively exaggerated adaptations for bipedalism.

## BACKGROUND AND OBJECTIVES

Back pain is perhaps the single greatest contributor to disability worldwide [1], with over 50% of people living in developed countries experiencing it at some point in their lives [2, 3]. As well as affecting the well-being of many people, back pain is economically burdensome [4]. For example, back pain-associated healthcare costs in the UK have been estimated to be between £3 billion and £12 billion per year [5, 6]. Back pain’s indirect economic impacts are also high. It has been estimated that in the USA alone companies lose as much as $7.4 billion per year due to back pain-related issues among workers [7]. Given its substantial individual and societal costs, investigating the causes of back pain is an important endeavour.

A few years ago, we proposed that a common spinal pathology, intervertebral disc herniation (IDH), may be linked to vertebral shape and ultimately to our lineage’s evolutionary shift from quadrupedalism to bipedalism [8]. IDH can be recognized on dry-bone vertebrae by the presence of Schmorl’s nodes, which are depressions on the vertebral endplate [9]. In the study, we compared the planar shape of the final thoracic and first lumbar vertebrae of humans (*Homo sapiens*), chimpanzees (*Pan troglodytes*), and orangutans (*Pongo pygmaeus*). The *H. sapiens* vertebrae were divided into two groups, one with Schmorl’s nodes and one without. We found that the Schmorl’s nodes-bearing *H. sapiens* vertebrae were closer in shape to the chimpanzee vertebrae than were the healthy *H. sapiens* vertebrae. When interpreting this finding, we took into account the fact that *Pan* and *Homo* share an ancestor to the exclusion of all other living taxa [10]. We hypothesized that the finding means that many people who develop IDH do so because their vertebrae are closer to the ancestral shape for the hominin lineage, and therefore are less well adapted for the stress placed on the spine during bipedalism. Recently, we tested this ‘Ancestral Shape Hypothesis’ with 3D data from extant humans and chimpanzees and fossil hominins and obtained results that are consistent with it [11].

**Figure 1.**
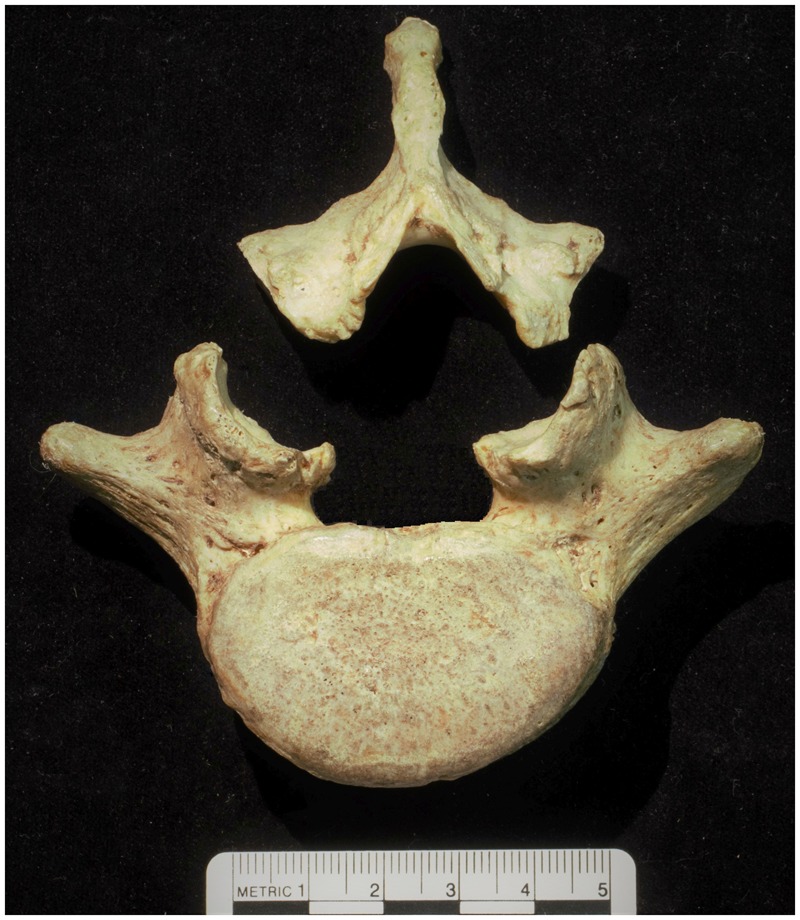
A human final lumbar vertebrae with bilateral spondylolysis

In this article, we report a study in which we attempted to use evolutionary theory and methods to shed light on another important spinal pathology—spondylolysis. Spondylolysis is a defect or abnormality of the pars interarticularis and the lamina and pedicle surrounding it [12] (Fig. 1). While medical researchers recognize several types of spondylolysis, most cases feature a cleft of the neural arch resulting from a fatigue fracture [12]. This type of spondylolysis is sometimes called ‘isthmic spondylolysis’ [13] or ‘chronic traumatic spondylolysis’ [12] but it is so much more common than the other types that it is usually just referred to ‘spondylolysis’. While spondylolysis can be asymptomatic, it often gives rise to lower back pain [14–17]. It is the leading cause of back pain in sub-adults [12] and can have significant impact on athletes [17]. For example, a 2004 study reported that almost 40% of athletes with pain related to spondylolysis withdrew from their athletic activities [18]. Non-human primate species do not seem to be prone to fatigue fracture-induced spondylolysis; to date spondylolysis has only been reported in non-human primate individuals with congenital abnormalities of the pars interarticularis [19].

Although the pathogenesis of spondylolysis is well understood, its aetiology is unclear. Several investigations of spondylolysis in human skeletal remains have suggested that vertebral shape may be an important factor in determining an individual’s propensity to develop the condition. For example, Grobler et al. [20], Miyake et al. [21], and Van Roy et al. [22] found an association between spondylolysis and facet morphology. Specifically, they found that the facets of the fourth and fifth lumbar vertebrae of individuals with spondylolysis were flatter and more coronally oriented than those of healthy individuals. In addition, the facets of individuals with spondylolysis were found to be smaller in the transverse direction than those of individuals without spondylolysis. So far, there is no biomechanical explanation for these associations [21].

Another way that vertebral shape may contribute to spondylolysis has been highlighted by studies carried out by Ward et al. [23–25]. These authors found that individuals with spondylolysis tend to have reduced mediolateral spacing between the zygapophyseal facets of adjoining vertebrae. Ward et al. [23–25] posited that this reduced inter-facet spacing leads to the articular processes of one vertebra directly contacting the pars interarticularis of the subjacent one, causing a fatigue fracture and ultimately spondylolysis.

Other researchers have found a link between spondylolysis and lumbar lordosis. Roussouly et al. [26] used radiographs to investigate the alignment of the spine and pelvis in individuals with and without spondylolysis and found an association between spondylolysis and increased lumbar lordosis. Masharawi et al. [27] found that individuals with spondylolysis tend to have fifth lumbar vertebrae that are more dorsally wedged (i.e. the ventral border of the vertebral body is craniocaudally taller than the dorsal border) than unaffected individuals. They suggested that the greater dorsal wedging increases lumbar lordosis, resulting in contact between the neural arches of the fourth and fifth lumbar vertebrae. It is this contact, Masharawi et al. [27] proposed, that causes the fractures that leads to spondylolysis.

Building on the finding that spondylolysis is associated with increased lumbar lordosis and the fact that lumbar lordosis is widely accepted to be an adaptation for bipedalism [28, 29], in the study reported here we tested the hypothesis that spondylolysis is the result of individuals having a number of vertebral traits that are effectively exaggerated adaptations for bipedalism. The idea here is that spondylolysis is the result of the opposite shape problem to IDH: whereas having vertebrae that are towards the ancestral end of the range of shape variation in *H. sapiens* increases the probability of the developing IDH, having vertebrae with traits that place them towards the highly derived end of the range of shape variation in *H. sapiens* increases the probability of developing spondylolysis. We will refer to this as the ‘Overshoot Hypothesis’ on the grounds that it posits that individuals with spondylolysis have developed the condition because their vertebrae have gone beyond the lineage-specific shape optimum for bipedalism as a result of random mutations and/or developmental problems.

Our study had three parts, all of which involved the use of 3D geometric morphometric techniques. In the first, we tested the prediction that the shape of *H. sapiens* final lumbar vertebrae with spondylolytic lesions is significantly different from the shape of healthy *H. sapiens* vertebrae. In the second, we compared the shape of *H. sapiens* final lumbar vertebrae with and without spondylolytic lesions to the shapes of the final lumbar vertebrae of chimpanzees, gorillas, and orangutans. The prediction tested in this part of the study was that the vertebrae of healthy *H. sapiens* should be closer in shape to those of the great apes than are *H. sapiens* vertebrae with spondylolytic lesions. In the third and final part of the study, we re-ran the previous analysis after adding a sample of final lumbar vertebrae from *H. sapiens* with Schmorl’s nodes in one or more of their vertebrae. Given what we found previously vis-à-vis Schmorl’s nodes and vertebral shape variation in *H. sapiens* [9], the prediction we tested in this analysis was that the groups should conform to the following pattern: the *H. sapiens* specimens with spondylolytic lesions should be least like those of the great apes; the *H. sapiens* specimens with Schmorl’s nodes should be most like those of the great apes; and the *H. sapiens* specimens without either pathology should fall between the other two groups of *H. sapiens* vertebrae.

## METHODOLOGY

Our sample comprised the final lumbar vertebra of 97 *H. sapiens*, 28 *P. troglodytes*, 29 *Po. pygmaeus*, and 22 *Gorilla gorilla* (Table 1). These individuals are curated at the Cleveland Museum of Natural History, the Natural History Museum Vienna, the Museum of Natural History Berlin, the University of Copenhagen, the University of Zurich, and the Smithsonian Institution National Museum of Natural History. All of the individuals were deemed to be fully adult based on epiphyseal fusion [30]. Twenty-one of the *H. sapiens* vertebrae exhibited bilateral spondylolysis, while another 26 were from individuals showing evidence of IDH in the form of Schmorl’s nodes [10] on one or more of their vertebrae. The remaining 50 *H. sapiens* vertebrae were from individuals without any visible spinal pathologies. Individuals were chosen for inclusion in the sample based on adequate preservation of the final lumbar vertebrae.

**Table 1.** Number of individuals measured for each human group and great ape species

Group/species	Females	Males	Total
*Homo sapiens*			
Spondylolytic	6	15	
Schmorl’s nodes-affected	12	14	
Healthy	24	26	97
*Pan troglodytes*	15	13	28
*Gorilla gorilla*	9	13	22
*Pongo pygmeaus*	13	16	29

**Figure 2.**
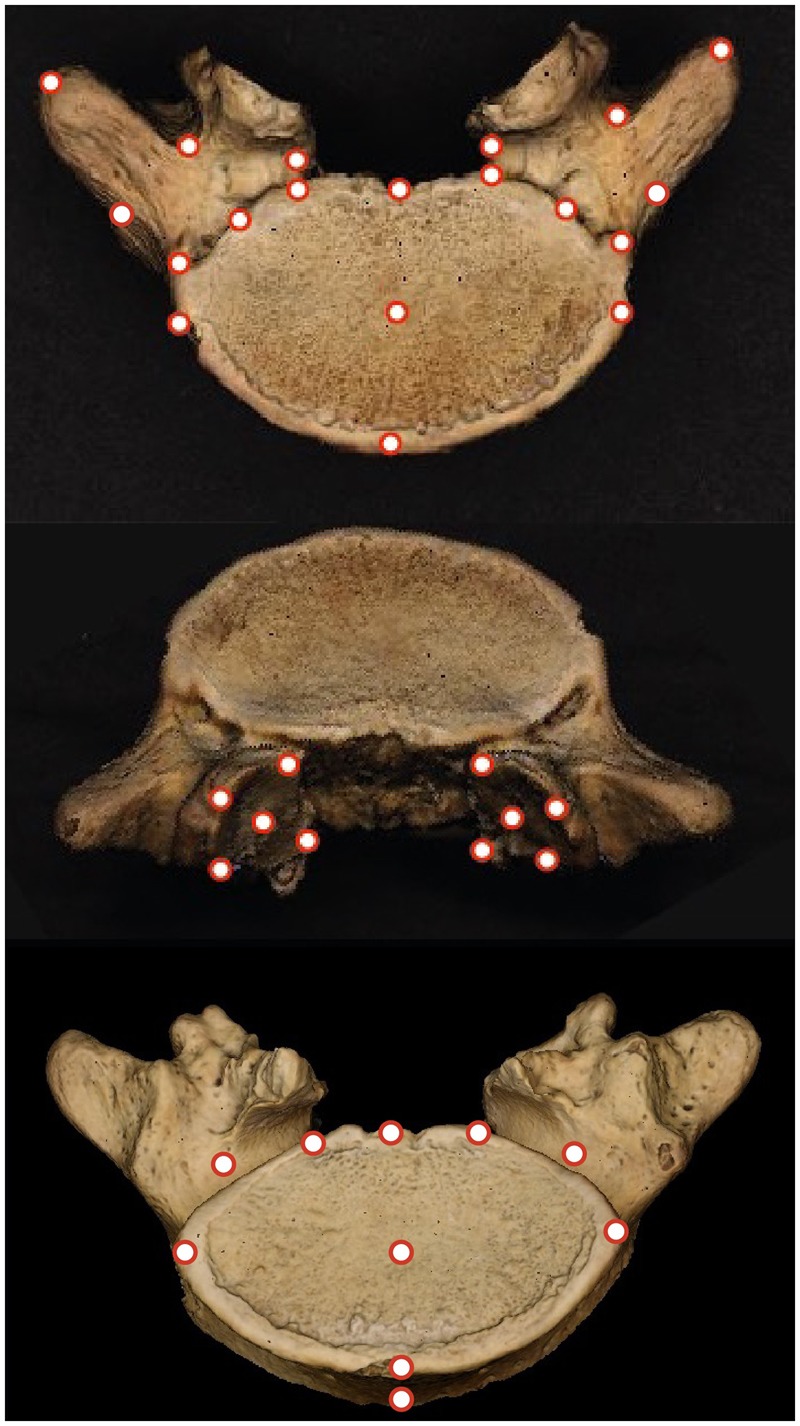
The location of the 39 landmarks used to capture the shape of the final lumbar vertebrae

We recorded the *x*, *y*, *z* coordinates of 39 landmarks using a Microscribe digitizing arm (Fig. 2). The landmarks were chosen to capture the shape of the body, pedicles, and superior zygapophyseal facets of each vertebra. Some of them were type II landmarks; others were type III landmarks [31]. Following Arnqvist and Martensson [32], each vertebra was digitized twice and the coordinates averaged. In a previous study, we demonstrated that the error associated with this set of landmarks after they have been recorded twice and averaged is unlikely to bias the results of analyses [33].

We removed the confounding effects of translation, rotation, size, and asymmetry by applying the approach outlined by Klingenberg et al. [34]. This involved reflecting and re-labelling the landmark coordinates, and then subjecting the data to generalized Procrustes analysis (GPA), which removes translational and rotational effects from landmark data and scales the configurations to centroid size [35]. Next, we removed asymmetry by calculating the average Procrustes coordinates between the original and reflected landmarks. The GPA was performed in Morphologika [36], and the average Procrustes coordinates were calculated in Excel.

Having minimized the confounding effects of translation, rotation, size, and asymmetry, we undertook three sets of analyses. In the first, we compared the spondylolytic and healthy *H. sapiens* specimens to see whether their shapes differ significantly. We began by subjecting the data to principal components analysis (PCA) and then implemented Baylac and Frieb’s [37] method for minimizing noise from principal components (PCs) that account for little variance. To identify low-information PCs, Baylac and Frieb’s [37] method progressively adds PCs into a discriminant function analysis (DFA) until cross-validation percentage (CVP) begins to drop; only PCs that contribute positively to the CVP are retained. Next, we applied DFA to the PCs to assess the ability of the data to classify the specimens as either spondylolytic or healthy [38]. Lastly, we subjected the PCs to a MANOVA to assess the statistical significance of the differences between the groups. We performed the PCA in Morphologika [36], the DFA in R [39], and the MANOVA in SPSS [40].

In the second set of analyses, we compared the spondylolytic and healthy *H. sapiens* vertebrae to the great ape vertebrae. The test prediction was that the healthy *H. sapiens* vertebrae should, on average, be closer in shape to the great ape vertebrae than are the spondylolytic *H. sapiens* vertebrae. As before, we subjected the data to PCA and then excluded uninformative PCs using Baylac and Frieb’s [37] method. Subsequently, we calculated the Procrustes distances between the means of the groups. The PCA was carried out in Morphologika [36], and the Procrustes distances were computed in R [39].

In the third set of analyses, we compared the spondylolytic *H. sapiens* vertebrae, the healthy *H. sapiens* vertebrae, the Schmorl’s nodes-affected *H. sapiens* vertebrae, and the great ape vertebrae. The test prediction was that the spondylolytic *H. sapiens* vertebrae should be least like the great ape vertebrae; the Schmorl’s nodes-affected *H. sapiens* specimens should be most like the great ape vertebrae; and the healthy *H. sapiens* specimens should be intermediate between the other two groups of *H. sapiens* vertebrae. The analyses were the same as those in the second set of analyses, as were the computer programs we employed.

## RESULTS

### Comparison of spondylolytic and healthy H. sapiens vertebrae

Eighteen PCs were retained by the noise reduction procedure. These PCs accounted for 91% of the shape variance.

The differences between the average shapes of the spondylolytic and healthy *H. sapiens* vertebrae were significant, according to the MANOVA (λ 0.293, *F* = 4.662, *P* < 0.0001). In the DFA, 87% of the specimens were correctly classified as either spondylolytic or healthy. Both of these results are consistent with the first test prediction, which was that the two groups of human vertebrae should be significantly different in terms of shape.

**Figure 3.**
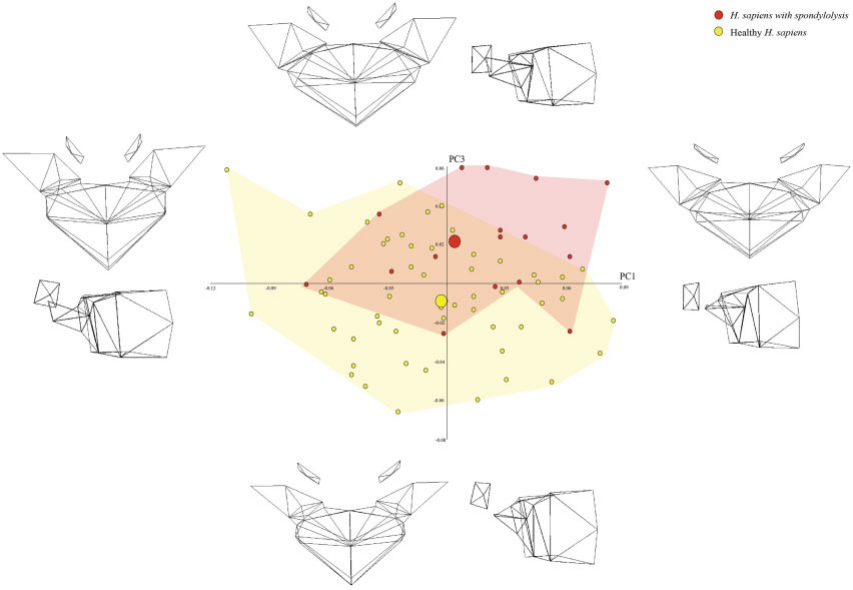
PCA scatter-plot depicting the shape variance on PC1 and PC3 when spondylolytic *H. sapiens* vertebrae are compared with healthy *H. sapiens* vertebrae. The wireframes illustrate the shape differences described by the PCs

The two groups were indistinguishable on most of the 18 PCs, but differences were apparent when PC3 (10% of shape variance) was plotted against PC1 (21% of shape variance). As can be seen in Fig. 3, the spondylolytic vertebrae were positioned more positively on PC3 than the healthy vertebrae.

The wireframes associated with Fig. 3 indicate that, compared to healthy *H. sapiens* vertebrae, spondylolytic *H. sapiens* vertebrae tend to have more pronounced dorsal wedging, and transverse processes that project more dorsally. They also have pedicles that project more dorsally, narrower inter-pedicle distances, and more coronally oriented zygapophyseal facets.

### Comparison of spondylolytic and healthy H. sapiens vertebrae with great ape vertebrae

The noise reduction procedure retained 23 PCs. These PCs accounted for 95% of the shape variance.

The Procrustes distances indicated that the average shape of the healthy *H. sapiens* vertebrae is closer to the average shape of the vertebrae of the three great ape species than is the average shape of the spondylolytic *H. sapiens* vertebrae (Table 2). This is consistent with the test prediction for this set of analyses, which was that the healthy *H. sapiens* vertebrae should be more similar in shape to the great ape vertebrae than are the spondylolytic *H. sapiens* vertebrae.

**Table 2.** Results of the second set of analyses

Comparison			Procrustes distance
Spondylolytic *H. sapiens*	vs	*P. troglodytes*	0.2184
	vs	*Po. Pygmaeus*	0.1936
	vs	*G. gorilla*	0.2297
Healthy *H. sapiens*	vs	*P. troglodytes*	0.1898
	vs	*Po. Pygmaeus*	0.1640
	vs	*G. gorilla*	0.2086

Procrustes distances were used to compare spondylolytic and healthy *H. sapiens* vertebrae with those of *P. troglodytes*, *Po. pygmaeus*, and *G. gorilla*. The Procrustes distances were generated from the 23 PCs that yielded the highest CVP.

**Figure 4.**
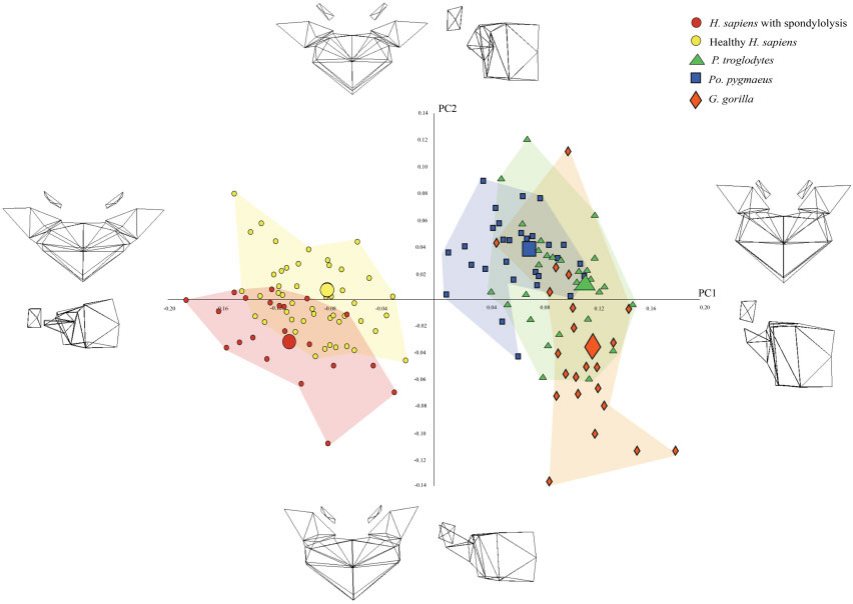
PCA scatter-plot depicting the shape variance on PC1 and PC2 when healthy and spondylolytic *H. sapiens* vertebrae are compared with those of *P. troglodytes*, *Po. pygmaeus*, and *G. gorilla*. The wireframes illustrate the shape differences described by the PCs

Once again, the groups overlapped substantially on most PCs. However, differences were apparent when PC2 (9% of shape variance) was plotted against PC1 (47% of shape variance). As can be seen in Fig. 4, the centre of the distribution of the spondylolytic *H. sapiens* vertebrae was located more negatively on both PCs than the centre of the distribution of the healthy *H. sapiens* vertebrae, and the latter was located more negatively than the centre of the distribution of the great ape vertebrae. This pattern is consistent with the test prediction.

The wireframes in Fig. 4 indicate that the differences between the spondylolytic and healthy *H. sapiens* vertebrae are the same as those described in the first set of analyses, but they also reveal three additional distinguishing traits. According to this set of wireframes, spondylolytic *H. sapiens* vertebrae also tend to have more concave inferior endplates, smaller inter-facet distances, and more caudally located facets than healthy *H. sapiens* vertebrae.

Expanding the comparison to include the great ape vertebrae adds a further important finding. The wireframes indicate that the traits that distinguish the spondylolytic *H. sapiens* vertebrae from the healthy *H. sapiens* vertebrae also distinguish the healthy *H. sapiens* vertebrae from the great ape vertebrae. Thus, the wireframes reveal that the spondylolytic *H. sapiens* vertebrae are more distant from the great ape vertebrae than are the healthy *H. sapiens* vertebrae. This implies that the spondylolytic *H. sapiens* vertebrae can be considered highly derived in relation to the traits and is consistent with the Overshoot Hypothesis.

### Comparisons of spondylolytic, Schmorl’s nodes-affected, and healthy H. sapiens vertebrae with those of great apes

Twenty-four PCs were retained by the noise reduction procedure. These accounted for 95% of the total shape variance.

The Procrustes distances indicated that, on average, the spondylolytic *H. sapiens* vertebrae were more different from the great ape vertebrae than were the healthy and Schmorl’s nodes-affected *H. sapiens* vertebrae, and that the latter were more similar to the great ape vertebrae than were the spondylolytic and healthy *H. sapiens* vertebrae (Table 3). This pattern is in line with the test prediction for this set of analyses, which was that the spondylolytic *H. sapiens* vertebrae should be least like the great ape vertebrae, the Schmorl’s nodes-affected *H. sapiens* vertebrae should be most like the great ape vertebrae, and the healthy *H. sapiens* vertebrae should fall between the other two groups of *H. sapiens* vertebrae.

**Table 3.** Results of the second set of analyses

Comparison			Procrustes distance
Spondylolytic *H. sapiens*	vs	*P. troglodytes*	0.2182
	vs	*Po. Pygmaeus*	0.1938
	vs	*G. gorilla*	0.2294
Schmorl’s nodes-affected *H. sapiens*	vs	*P. troglodytes*	0.1823
	vs	*G. gorilla*	0.2000
	vs	*Po. pygmaeus*	0.1589
Healthy *H. sapiens*	vs	*P. troglodytes*	0.1894
	vs	*G. gorilla*	0.2079
	vs	*Po. Pygmaeus*	0.1639

Procrustes distances were used to compare spondylolytic *H. sapiens* vertebrae, the vertebrae of Schmorl’s nodes-affected *H. sapiens*, and healthy *H. sapiens* vertebrae to *P. troglodytes*, *Po. pygmaeus*, and *G. gorilla* vertebrae. Procrustes distances were generated from 24 PCs with the highest CVP.

**Figure 5.**
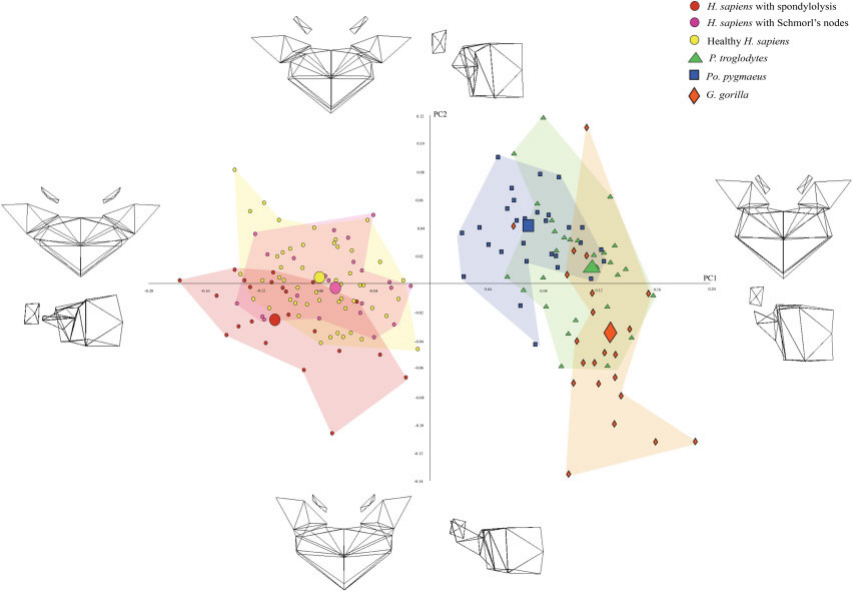
PCA scatter-plot depicting the shape variance on PC1 and PC2 when spondylolytic*,* Schmorl’s nodes-affected, and healthy *H. sapiens* are compared with those of *P. troglodytes*, *G. gorilla*, and *Po. pygmeaus*. The wireframes illustrate the shape differences described by each PC

As with the previous two sets of analyses, there was no distinction between groups on most PCs. Differences could be discerned when PC2 (9% of shape variance) was plotted against PC1 (47% of shape variance), however. As shown in Fig. 5, the three *H. sapiens* groups were clearly separated from the great apes on PC1 but not on PC2. Of the three *H. sapiens* groups, the Schmorl’s nodes-affected *H. sapiens* are the closest to the great apes, the healthy *H. sapiens* were the next closest, and the spondylolytic *H. sapiens* were the most distant. This pattern is also consistent with the test prediction.

The wireframes indicate that the traits that distinguish *H. sapiens* vertebrae from great ape vertebrae and those that distinguish spondylolytic *H. sapiens* vertebrae from healthy *H. sapiens* vertebrae are the same as the traits identified in the previous analysis. In addition, the wireframes show that shape differences that distinguish Schmorl’s nodes-affected *H. sapiens* vertebrae from those of spondylolytic and healthy *H. sapiens* are the same as those that distinguish great ape vertebrae from spondylolytic and healthy *H. sapiens* vertebrae. Specifically, the wireframes indicate that, compared with both the spondylolytic and healthy *H. sapiens* vertebrae, the Schmorl’s nodes-affected *H. sapiens* vertebrae tend to have less pronounced dorsal wedging; transverse processes that project more laterally; laterally projecting pedicles; zygapophyseal facets that are more cranially located; narrower inter-pedicle distances; narrower inter-facet distances; and more concave inferior endplates. In all these traits, the Schmorl’s nodes-affected *H. sapiens* vertebrae are closer in shape to the great ape vertebrae than are the other two groups of *H. sapiens* vertebrae. This pattern is consistent with the test prediction as well.

## DISCUSSION

### Summary of results

In the study reported here, we tested the Overshoot Hypothesis for spondylolysis, which holds that having vertebrae that are towards the highly derived end of the range of shape variation in *H. sapiens* predisposes individuals to develop spondylolysis. To test the hypothesis, we compared the 3D shape of healthy and pathological *H. sapiens* final lumbar vertebrae to each other and to great ape final lumbar vertebrae. Our analyses yielded three main findings. First, we found that the average shape of *H. sapiens* vertebrae with spondylolysis is significantly different from the average shape of healthy *H. sapiens* vertebrae. We also found that the healthy *H. sapiens* specimens in our sample were closer in shape to the great ape specimens than were the spondylolytic *H. sapiens* specimens. Lastly, we found that the spondylolytic vertebrae fell at the opposite end of the range of shape variation within *H. sapiens* to the vertebrae of individuals with evidence of having experienced IDH, which we have previously shown is associated with possession of an ancestral vertebral shape [8, 9]. Taken together, these findings suggest that individuals who suffer from spondylolysis do indeed have vertebrae that lie at the highly derived end of the range of variation within *H. sapiens*. As such, they support the Overshoot Hypothesis.

### Vertebral traits associated with spondylolysis

Three of the traits that our analyses indicate are associated with spondylolysis have been identified in previous studies—increased dorsal wedging, narrower inter-facet distances, and zygapophyseal facets that are more coronally oriented [23–25, 27]. The analyses also identified five traits that have not previously been linked with spondylolysis, to the best of our knowledge. These are dorsally projecting pedicles, narrower inter-pedicle distances, caudally located zygapophyseal facets, concave inferior endplates, and dorsally projecting transverse processes.

It seems likely that two of the newly identified traits—narrower inter-pedicle distance and dorsal projection of the pedicles—are related to narrow inter-facet distances, which Ward et al. [23–25] found to be associated with spondylolysis. Both the projection of the pedicles and the inter-pedicle distance can be expected to influence the width of the neural arch and this in turn can be expected to influence the distance between facets. Specifically, pedicles that are closer together and project dorsally (rather than flaring laterally) can be expected to contribute to a narrower neural arch and this can be expected to lead to narrower inter-facet distances.

There is also reason to think that the more caudal location of the zygapophyseal facets may increase the risk of spondylolysis by reducing spacing between the inferior facets of the fourth lumbar vertebra and the superior facets of the subjacent, fifth lumbar vertebra. Insufficient subjacent spacing between the neural arches of the fourth and fifth lumbar vertebrae likely would result in crowding of the joints and lead to increased contact between the inferior facets of the fourth vertebra and the pars interarticularis of the fifth lumbar vertebra. This can be expected to increase the probability of fatigue fractures that can eventually lead to spondylolysis.

The shape of the vertebral endplates is thought to play a role in dispersing compressive stress in the lower lumbar vertebrae [41, 42]. Liu et al. [41] found that endplates with shallower concavities decrease the amount of stress placed on the zygapophyseal facets and neural arch, while He et al. [42] found that less concave endplates are better suited to withstand compressive strains on the vertebral disc and body. One corollary of these findings is that the facets and neural arches of vertebrae with deeper concavities, such as the spondylolytic vertebrae in our study, may have more stress placed on them. The increased stress on these elements can be expected to increase the probability of fatigue fractures and ultimately spondylolysis.

At the moment, the more dorsal orientation of the transverse processes does not appear to be causally related to spondylolysis. Unlike the other vertebral elements under consideration, the lumbar transverse processes do not seem to play a significant role in withstanding stress or allowing adequate spacing between vertebrae [43]. Their primary function is to serve as attachment sites for the spinae muscles, which maintain lordosis during bipedal posture and gait [43–45]. A dorsal projection of the lumbar transverse processes would increase the lever arms of the erector spinae muscles [46–48], increasing the ability of the muscles to maintain lumbar lordosis during bipedalism [49, 50]. While this is consistent with the idea that vertebrae with spondylolysis tend to have highly derived traits, there is no obvious biomechanical reason why it should increase the propensity to develop spondylolysis. Thus, we suggest that, for the time being, the more dorsal orientation of the transverse processes should be considered to be correlated with spondylolysis but not causally related to it.

### Future directions

Given the results of the study reported here, there are three obvious potential avenues for research in the future. First, it would be useful to test the hypothetical biomechanical links between the newly identified vertebral shape traits and spondylolysis. This could be accomplished using medical imaging technology and 3D morphometrics to investigate the interaction between bipedalism and vertebral shape. Such a study might be able to identify patterns in human posture and locomotion that interact with vertebral shape and spinal musculature in such a way that they predispose individuals to spondylolysis. Potentially this could help clinicians and sports therapists identify individuals who are at a greater risk of experiencing fatigue fractures in their lower lumbar vertebrae. Athletes would be an obvious focus for such a study.

Second, it would be useful to repeat the analyses presented here with vertebrae of extinct hominins, such as *Australopithecus* and *Paranthropus*, included in the sample. Plomp et al. [11] carried out a similar study in which they tested the Ancestral Shape Hypothesis by comparing the shape of vertebrae from humans with and without Schmorl’s nodes with those of several extinct hominins. They found that vertebrae from humans with Schmorl’s nodes were generally closer in shape to the extinct hominin vertebrae than were healthy human vertebrae. They interpreted these findings as supporting the Ancestral Shape Hypothesis. The Overshoot Hypothesis could be tested in the same way. If such a study were to find that vertebrae of extinct hominins share more similarities in shape with vertebrae of healthy *H. sapiens* and great apes than with spondylolytic *H. sapiens* vertebrae, this would provide further support for the hypothesis.

Lastly, the findings of the present study and those we obtained in our previous studies [8, 11] show that analysing a spinal pathology within an evolutionary framework can provide valuable insight into the pathology’s aetiology. Given this, it would be sensible to investigate whether other spinal pathologies are associated with particular vertebral shapes and whether those shapes lie at one end or the other of the range of vertebral shape variation in *H. sapiens*. Indeed, in view of the fact that the shift from quadrupedalism to bipedalism affected multiple regions of the skeleton, it would be sensible to go beyond spinal pathologies and examine other skeletal pathologies.

## CONCLUSIONS

The present study sought to shed light on the aetiology of the spinal pathology known as spondylolysis. Using 3D data recorded on the final lumbar vertebrae of three groups of *H. sapiens* and three great ape species, we tested the Overshoot Hypothesis, which proposes that people develop spondylolysis because their vertebrae are at the highly derived end of the range of shape variation within *H. sapiens*. The results we obtained were clear-cut. We found that *H. sapiens* vertebrae with spondylolysis are significantly different in terms of shape from healthy *H. sapiens* vertebrae, and that *H. sapiens* vertebrae with spondylolysis are more distant from great ape vertebrae than are healthy *H. sapiens* vertebrae. We also found that *H. sapiens* vertebrae with spondylolysis are at the opposite end of the range of variation than vertebrae with Schmorl’s nodes, which previous studies have revealed are at the ancestral end of the range of variation [8, 9]. Together, these three findings strongly support the Overshoot Hypothesis. More generally, the study adds weight to the idea that where an individual’s vertebrae sit on the spectrum of vertebral shape variation within *H. sapiens* plays a role in their propensity to develop different spinal pathologies [8, 11]. This could have important implications for the prevention and management of back pain.
